# Obituary

**Published:** 1847-02

**Authors:** 


*Obituary.*—Died at South Wales, Erie Co., on the 8th ult., Dr. Ira G. Watson, aged 56 years. Dr. Watson had been a resident of Wales for 35 years, and was greatly esteemed as a judicious practitioner of Medicine, and as an upright, estimable man.

